# Untargeted Metabolomics Analysis Revealed Lipometabolic Disorders in Perirenal Adipose Tissue of Rabbits Subject to a High-Fat Diet

**DOI:** 10.3390/ani11082289

**Published:** 2021-08-03

**Authors:** Siqi Xia, Jiahao Shao, Mauricio A. Elzo, Tao Tang, Yanhong Li, Tianfu Lai, Mingchuan Gan, Yuan Ma, Xianbo Jia, Songjia Lai, Jie Wang

**Affiliations:** 1 College of Animal Science and Technology, Sichuan Agricultural University, Chengdu 611130, China; xiasiqi2020@163.com (S.X.); shaojh1997@163.com (J.S.); m18483220592@163.com (T.T.); lyh81236718@163.com (Y.L.); tf.lai@foxmail.com (T.L.); ganmingchuan1998@163.com (M.G.); manima0916@163.com (Y.M.); 2 Department of Animal Science, University of Florida, Gainesville, FL 32611, USA; maelzo@ufl.edu; 3 Farm Animal Genetic Resources Exploration and Innovation Key Laboratory of Sichuan Province, Sichuan Agricultural University, Chengdu 611130, China; jaxb369@sicau.edu.cn (X.J.); laisj5794@163.com (S.L.)

**Keywords:** high-fat diet, lipometabolic, metabolomics, perirenal adipose tissue (PAT), rabbit

## Abstract

**Simply Summary:**

A high-fat diet is widely recognized as a significant modifiable risk for metabolic diseases. In this study, untargeted metabolomics, combined with liquid chromatography and high-resolution mass spectrometry, was used to evaluate perirenal adipose tissue metabolic changes. Our study revealed 206 differential metabolites. These metabolites were mainly associated with the biosynthesis of unsaturated fatty acids, the arachidonic acid metabolic pathway, the ovarian steroidogenesis pathway, and the platelet activation pathway. Our study revealed that a high-fat diet causes significant lipometabolic disorders; these metabolites may inhibit oxygen respiration by increasing adipocytes cells and density, cause mitochondrial and endoplasmic reticulum dysfunction, produce inflammation, and finally lead to insulin resistance, thereby increasing the risk of Type 2 diabetes, atherosclerosis, and other metabolic syndromes.

**Abstract:**

A high-fat diet (HFD) is widely recognized as a significant modifiable risk for insulin resistance, inflammation, Type 2 diabetes, atherosclerosis and other metabolic diseases. However, the biological mechanism responsible for key metabolic disorders in the PAT of rabbits subject to HFD remains unclear. Here, untargeted metabolomics (LC-MS/MS) combined with liquid chromatography (LC) and high-resolution mass spectrometry (MS) were used to evaluate PAT metabolic changes. Histological observations showed that the adipocytes cells and density of PAT were significantly increased in HFD rabbits. Our study revealed 206 differential metabolites (21 up-regulated and 185 down-regulated); 47 differential metabolites (13 up-regulated and 34 down-regulated), comprising mainly phospholipids, fatty acids, steroid hormones and amino acids, were chosen as potential biomarkers to help explain metabolic disorders caused by HFD. These metabolites were mainly associated with the biosynthesis of unsaturated fatty acids, the arachidonic acid metabolic pathway, the ovarian steroidogenesis pathway, and the platelet activation pathway. Our study revealed that a HFD caused significant lipometabolic disorders. These metabolites may inhibit oxygen respiration by increasing the adipocytes cells and density, cause mitochondrial and endoplasmic reticulum dysfunction, produce inflammation, and finally lead to insulin resistance, thus increasing the risk of Type 2 diabetes, atherosclerosis, and other metabolic syndromes.

## 1. Introduction

In recent years, the occurrence rate of obesity has fleetly increased, which poses a risk for many medical diseases, causing concern for many public and health-related professionals. Obesity is a serious medical, social, and economic problem that has caused millions of disabilities, concomitant diseases, and deaths [1,2]. The prevalence of obesity in humans is widespread across all ages and both sexes and can be attributed to the interaction between the environment and physiological factors [3,4]. Nowadays, the universality of a HFD is one of the major contributors to the development of obesity. Moreover, many analyses across various species (rats, mice, and pigs) have indicated that HFD is associated with multiple metabolic syndromes, such as insulin resistance, Type 2 diabetes, cardiovascular disease, fatty liver, hypertension, Alzheimer’s disease, and cancer [5,6,7,8,9,10,11,12]. PAT is a kind of white adipose tissue that supports triglyceride (TG) storage for energy demands and endocrine function. Broadly, studies have revealed that PAT plays a significant role in controlling lipid mobilization and reproductive function as well as modulating multiple metabolic pathways [13,14]. Accumulated evidence shows that the main mechanisms leading to these metabolic diseases include endoplasmic reticulum stress and mitochondrial dysfunction [15], excessive accumulation of metabolites in adipose tissue, imbalance of energy supply and metabolic homeostasis [16], reduction of reverse cholesterol transport [17], aggravation of inflammation and reduction of insulin sensitivity [18]. Above all, one of the most significant and destructive complications is abnormal lipid metabolism, which will definitely worsen in the future [19].

With the popularity of metabolite research, the detection methods of metabolites have also been innovated. Metabonomics is usually used as a tool to discover biomarkers, which can analyze metabolites in biological fluids, tissues, and cells [20]. Moreover, untargeted metabolomics analysis is a kind of metabonomics that can detect and analyze all small molecule metabolites simultaneously without bias. Untargeted metabolomics analysis can evaluate metabolites in detail, explain the categories of metabolites, and examine the relationship between related metabolites in multiple ways [21]. Metabolites maintain homeostasis in response to adverse biological responses. Previous studies have also shown that the use of untargeted metabolomics analysis provides a basis for metabolic syndrome in HFD-fed rats [22,23]. Therefore, untargeted metabolomics is used to identify the phenotype-related metabolites and metabolic pathways to explain the specific functions of metabolites and to understand the physiological effects.

Rabbits are economically important domestic animals raised primarily as a source of animal protein, more recently used as a practical model for obesity-related studies [24]. A previous study reported that the subcutaneous adipose tissue of HFD-fed New Zealand white rabbits after 5 or 10 weeks plays a significant role in obesity-associated systemic low-grade inflammation [25]. Additionally, there was a study that evaluated changes in blood vessel function, using a HFD-rabbit model [26]. However, the overall metabolic change of PAT in rabbits fed a HFD has yet to be elucidated. Thus, to gain further understanding of the molecular consequences of obesity, we investigated the metabolic change in PAT from the obese rabbit induced by a HFD by using untargeted metabolomics.

## 2. Materials and Methods

### 2.1. Ethics Statement

This study was approved by and conducted in strict accordance with the ethical standards of the Institutional Animal Care and Use Committee of the College of Animal Science and Technology, Sichuan Agricultural University, Sichuan, China.

### 2.2. Animals and Experimental Design

A total of 16 female Tianfu black rabbits (35 days of age) at the teaching farm of the Sichuan Agricultural University were randomly divided into two groups: a control group fed a SND (*n* = 8) and an experimental group fed a HFD (SND plus 10% lard, *n* = 8). Detailed information on the feeding procedures can be found in a previous study [27]. Briefly, rabbits from both groups were fed twice a day with free access to water and under a light/dark cycle of 12 h per day. The room temperature was about 22 to 26 °C. According to the previous research method, the animals were classified as obese [27]. In short, the body weight, body length, bust length and adipose tissue weight were used as the criteria for selecting obese rabbits. After eliminating the substandard rabbits, 6 rabbits were selected from the HFD (*n* = 6) and SND (*n* = 6) groups, respectively, for sampling. After being fed for 5 weeks, the selected rabbits were humanely slaughtered by electrical stunning with exsanguinations for sampling. The rabbit PAT samples were rapidly collected after rabbits were euthanized and stored in Eppendorf tubes at −80 °C for subsequent analysis.

### 2.3. Histological Examination

In order to examine the histological changes of PAT, selected rabbits were humanely slaughtered, and PAT was stained with hematoxylin-eosin. In brief, the tissue samples were fixed with a 10% neutral formaldehyde fixator for about 24 h and then washed with clean water. After that, the tissue samples were dehydrated, embedded in paraffin, and stained with hematoxylin-eosin. Then, the PAT sections (6 to 8 μm) were collected using a microtome (RM2235, Leica, Nussloch, Germany). Furthermore, an alternative microscope (DM1000, Leica, Nussloch, Germany) was used to capture images at a 200 × field of view. In the end, PAT slices of rabbits from the two groups were analyzed by ImageJ software (National Institutes of Health). We used ImageJ (available at https://imagej.nih.gov/ij/ accessed on 22 July 2021) to quantitatively analyze the staining sections of perirenal adipose tissue in HFD and SND groups, respectively. Briefly, we calculated the PAT cells with more than 4.38 pixels, and calculated the 5 parts (upper, lower, left, right and central regions) of each staining section. Six cells were randomly selected from each region, with a total of 30 cells [28]. Finally, the standard error of mean was calculated, and the unpaired *t*-test was used to compare the differences of PAT between HFD and SND.

### 2.4. Sample Preparation

Approximately 100 mg of the PAT sample from each rabbit was ground into powder in liquid nitrogen. Homogenized samples were resuspended and vortexed by adding a prepared mixture composed of 80% methanol and 0.1% formic acid. Then, the samples were placed on ice and incubated for 5 min, followed by centrifugation at 15,000× *g* and 4 °C for 5 min. The methanol concentration of the supernatants was reduced to 53% by LC-MS/MS grade water. After the samples were transferred to a new Eppendorf tube, they were centrifuged at 15,000× *g* and 4 °C for 10 min. Lastly, the supernatants were incorporated into the LC-MS/MS system to conduct the LC-MS/MS analysis [29].

### 2.5. Ultra-High Performance Liquid Chromatography-Tandem Mass Spectrometry (UHPLC-MS/MS) Analysis

Chromatographic separation of differential metabolites was accomplished by injecting PAT samples into a Hypesil Gold HPLC column (2.1mm × 100 mm, 1.9 μm) (Thermo Fisher, Waltham, MA, USA) using a Vanquish UHPLC System (Thermo Fisher, Bremen, Germany) coupled with a Q Exactive HF-X Hybrid Quadrupole-Orbitrap Mass Spectrometer (Thermo Fisher, Germany) located at Novogene Co., Ltd. (Beijing, China). In detail, the Hypesil Gold HPLC column was kept at 40 °C with a flow rate of 0.2 mL/min using a 17 min linear gradient. The eluent B (Methanol) was the same for both positive and negative polarity modes. However, the eluent A differed for the positive (0.1% FA in Water) and negative (5 mM ammonium acetate, pH 9.0) polarity modes. The solvent gradient was set as follows: 2% B, 1.5 min; 2 to 100% B, 12.0 min; 100% B, 14.0 min; 100 to 2% B, 14.1 min; 2% B, 17 min. The Q Exactive HF-X Hybrid Quadrupole-Orbitrap Mass Spectrometer was operated in both positive and negative electron spray ionization (ESI+)/(ESI−) modes with a spray voltage of 3.2 kV. Moreover, the settings were 320 °C for the capillary temperature, 40 arb for the sheath gas flow rate, and 10 arb for the aux flow rate.

### 2.6. Data Processing and Analysis

The raw data files generated by UHPLC-MS/MS were processed with Compound Discoverer 3.1 (CD 3.1, Thermo Fisher) to determine the peak value for each metabolite. The detailed parameters were set as follows: (a) the retention time tolerance was 0.2 min; (b) the actual mass tolerance was 5 ppm; (c) the signal intensity tolerance was 30%; (d) the signal ratio was 3; and (e) the minimum intensity was 100,000. Then, the peak values were matched with the mzCloud (https://www.mzcloud.org/ accessed on 24 August 2020), mzVault, and MassList databases to obtain the exact and relative quantitative results. To better observe the inter-group distributions and otherness, the program metaX was used for PCA and PLS-DA. The quality of the PLS-DA models was assessed, using R2X, R2Y, and Q2. R2X and R2Y are fractions of sum of squares explained by a given principal component. Q2 represents the predictive ability of the PLS-DA model. Permutation tests with 200 permutations were used to validate the models.

### 2.7. Identification and Analysis of Metabolites

Differently expressed metabolites were identified based on the value of VIP, the *p*-value, and fold change (FC). In detail, the metabolites with variable importance in projection (VIP) > 1, *p*-value < 0.05, and FC ≥ 2 or FC ≤ 0.5 were considered to be differential metabolites. Moreover, volcano plots were used to filter metabolites of interest, using log2 (FC) and -log10 (*p*-value). Differential metabolites were annotated with the Human Metabolome Database (HMDB, https://hmdb.ca/metabolites, accessed on 24 August 2020), and LIPID MAPS (http://www.lipidmaps.org/ accessed on 24 August 2020) databases, respectively, to obtain a systematized overview. The differential metabolites-related metabolic pathways and physiological functions were explored by using the Kyoto Encyclopedia of Genes and Genomes (KEGG) database (https://www.genome.jp/kegg/pathway.html, accessed on 24 August 2020). A differential metabolic pathway was considered to be significantly enriched when the observed frequency of a metabolite (x/*n*) > expected frequency of a metabolite (y/*n*), and *p* value < 0.05. Clustering heat maps were plotted with R package Pheatmap (https://cran.r-project.org/web/packages/pheatmap/pheatmap.pdf, accessed on 24 August 2020).

### 2.8. Statistical Analysis

In this study, statistical software available in R (R version R-3.4.3) (MathSoft, Auckland, New Zealand), Python (Python 2.7.6 version) (The Python Software Foundation, Amsterdam, The Netherlands) and CentOS Linux (CentOS release 6.6) (Ctrl IQ) were used to analyze the metabolic data. A univariate *t*-test was applied to calculate the statistical significance, and only *p*-values < 0.05 were considered statistically significant. The *p* values were adjusted for multiple hypotheses by using the Benjamini–Hochberg procedure, controlling for the false discovery rate (FDR) (https://cran.r-project.org, accessed on 30 July 2021).

## 3. Results

### 3.1. PAT Histological Observations

**Figure 1:**
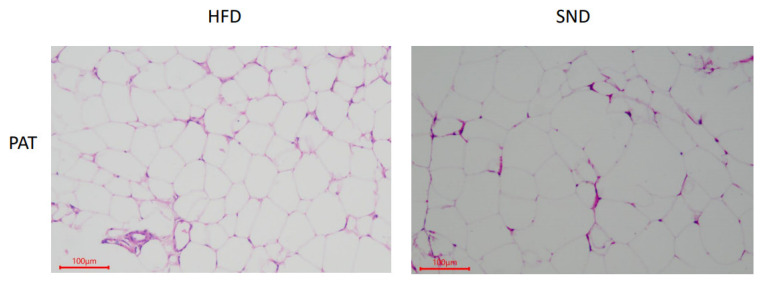
Hematoxylin-eosin staining of PAT in female rabbits fed SND (*n* = 6) and HFD (*n* = 6).

The hematoxylin-eosin-stained PAT samples in the SND and HFD groups showed a normal cell structure; however, a difference in the cell size and number was observed (Figure 1). Compared with the SND group, the number of PAT cells in the HFD was significantly increased (100.3 ± 1.453 vs. 77.33 ± 2.906, *p* = 0.0021); however, the area of PAT cells was significantly decreased (779.45 ± 30.822 μm vs. 1759.59 ± 100.502 μm, *p* < 0.0001).

### 3.2. Quality Control of Metabolomics Data

**Figure 2:**
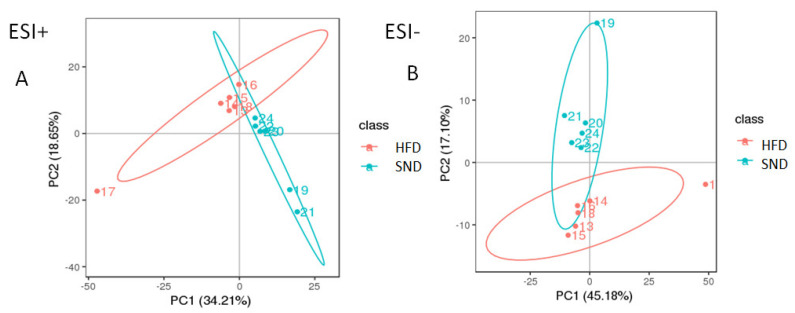

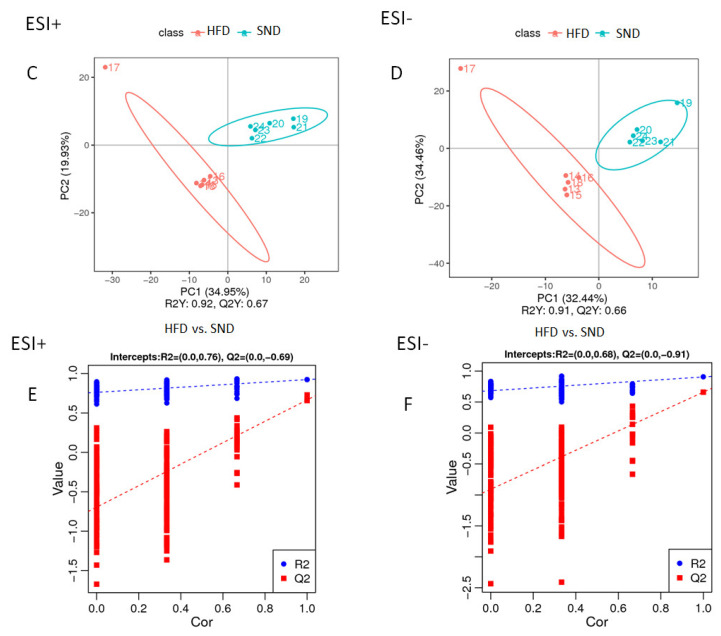
Quality control of metabolomics data. PCA score plots for SND (*n* = 6) and HFD (*n* = 6) groups in the positive (**A**) and negative (**B**) ion modes. PLS-DA score plots for SND (*n* = 6) and HFD (*n* = 6) groups in the positive (**C**) and negative (**D**) ion modes. Permutation tests from PLS-DA models for SND (*n* = 6) and HFD (*n* = 6) groups in the positive (**E**) and negative (**F**) ion modes.

The Pearson correlation coefficients between the positive and negative quality control (QC) samples are shown in Figure S1. The Pearson correlation coefficient between the QC samples is higher than 0.991 for both ESI+ and ESI−, indicating that the stability of detection and quality of data are excellent. The PCA score plots show that the distributions of PAT metabolites from the SND and HFD groups differ (Figure 2A,B). All the PLS-DA score plots for the SND and HFD groups (Figure 2C,D) are within the 95% confidence interval, except for the 17th and 19th samples. Samples #17 and #19 are not within the confidence interval. For the following analysis, these two samples are not included in the experimental analysis. The values of the PLS-DA statistics are R2Y = 0.92 and Q2 = 0.67 for the ESI+ data, and R2Y = 0.91, Q2 = 0.66 for the ESI− data. These R2Y and Q2 values indicate that the PAT metabolic differences between SND and HFD rabbits were quite significant. Permutation tests were used to prevent over-fitting of the PLS-DA models. The validation included 200 random permutation tests, which generated intercepts of R2 = 0.76 and Q2 = −0.69 for the ESI+ data and R2 = 0.68 and Q2 = −0.91 for the ESI− data (Figure 2E,F), indicating that the PLS-DA models are credible and without over-fitting. Thus, the PLS-DA models show an excellent predictive ability and reliability to determine significant PAT metabolic disturbances in HFD rabbits.

### 3.3. Differential Metabolites Analysis

**Figure 3:**
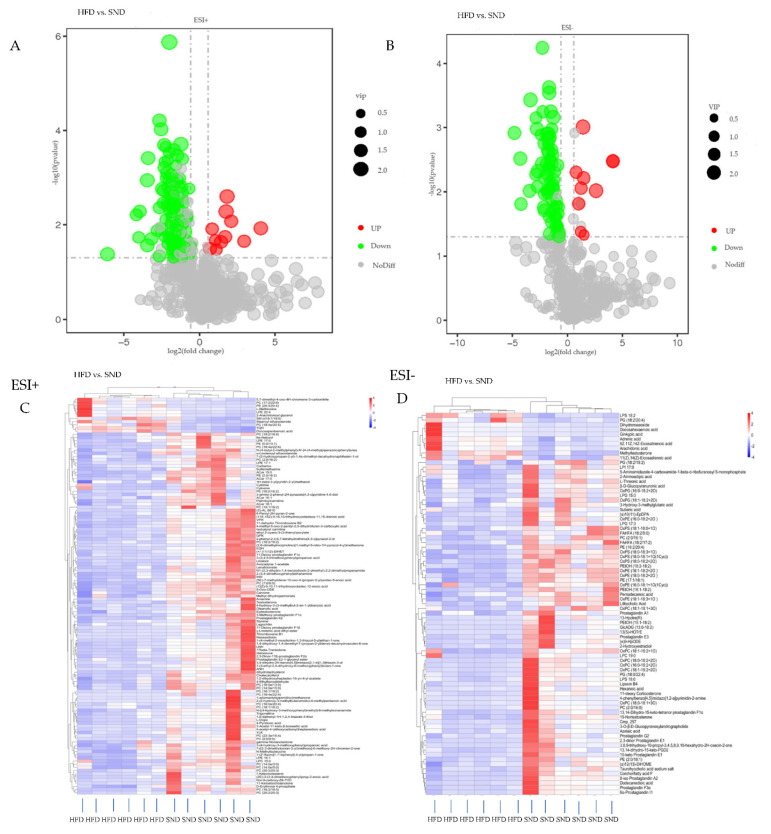
Differential metabolites analysis. Volcano maps showing the overall distribution of differential metabolites in rabbit groups fed SND (*n* = 6) and HFD (*n* = 6) in the positive (**A**) and negative (**B**) ion modes. Red circles represent significantly higher numbers of metabolites, and green circles indicate significantly lower numbers of metabolites in HFD (*n* = 6) than in SND (*n* = 6) rabbit groups. Heat map representing a hierarchical clustering of positive (**C**) and negative (**D**) ion mode of differential metabolites between HFD and SND. Shades of red and blue represent an increase or reduction of a metabolite, respectively, relative to median metabolite levels (see color scale). A = HFD, a = SND.

The VIP of the first principal component of the PLS-DA model combined with the *p* value was used to ascertain metabolites with differential expression in the SND and HFD groups. The thresholds were VIP > 1.0, FC > 1.5 or FC < 0.665, and *p* value < 0.05 [30,31,32]. The value of FDR is shown in Tables S1 and S2. A total of 206 metabolites were detected, of which 21 were up-regulated, and 185 were down-regulated (Tables S1 and S2). The volcano maps in Figure 3A,B visually show the overall distribution of the differential metabolites. Based on KEGG, HMDB and LIPID MAPS databases, a total of 50 metabolic pathways were detected in the HFD group, compared with the SND group in the two modes, of which fatty acids, phospholipids, and sterol lipids were identified as the main metabolites (Tables S3 and S4). Then, on this basis, the metabolites chosen as potential biomarkers to help explain metabolic disorders caused by HFD were those with VIP values above 1 and a *p* value below 0.05 [33]. A total of 47 metabolites were detected, of which 13 were significantly up-regulated, and 34 were significantly down-regulated (Table 1). In addition, hierarchical clustering analysis was conducted on the obtained metabolites of the two groups to obtain the differences of metabolic expression patterns between and within the same comparison group. So, the cluster heat map also showed the distribution of differential metabolites between the HFD and SND (Figure 3C,D). These results further illustrate that the metabolism of PAT was disturbed by HFD.

### 3.4. Metabolic Pathway Analysis

**Figure 4:**
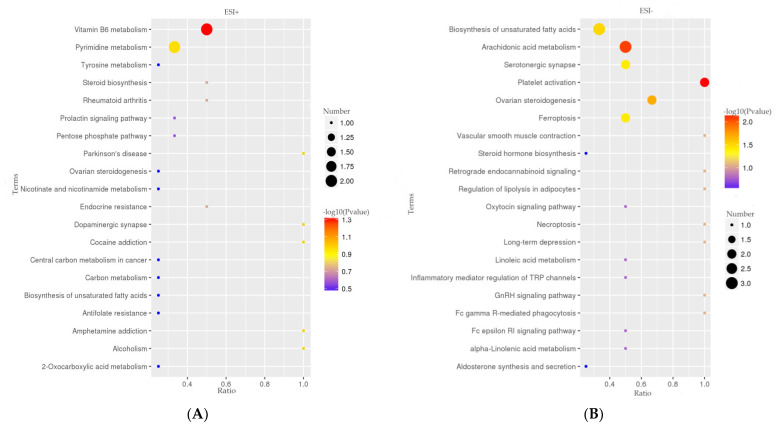

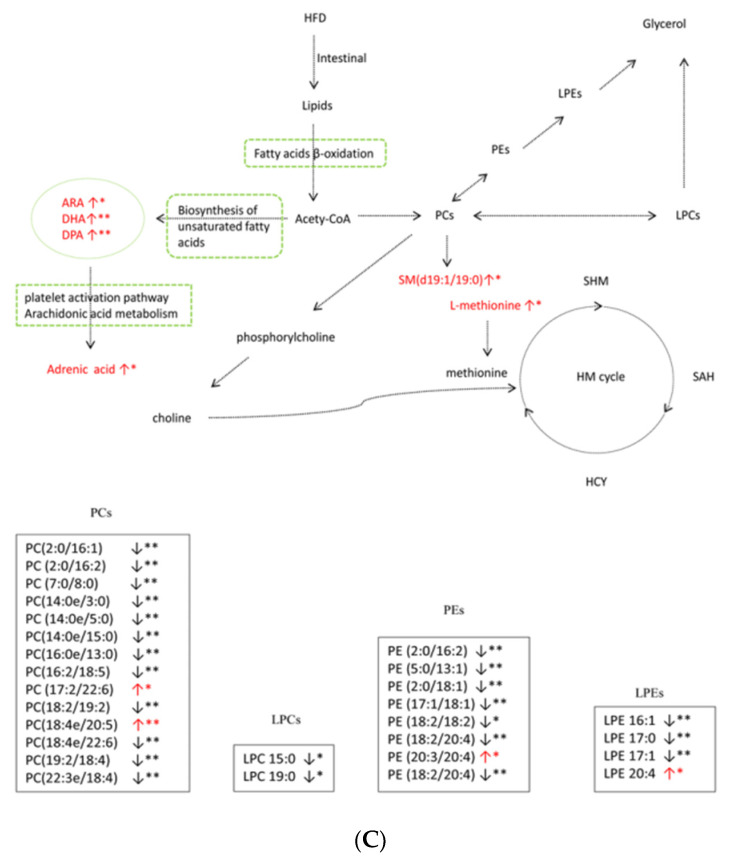
Metabolic pathway analysis. KEGG positive (**A**) and negative (**B**) pathway enrichment plots obtained with MetaboAnalyst. Metabolic pathways are represented by circles, circle size and color shade are based on pathway impact and *p* value (red being the most significant). (**C**) The network of metabolic pathways associated with the lipid cycle. Metabolite levels are shown in color: red represents increased levels, and black represents decreased or undetected levels. Trend and *p* value (HFD vs. SND): * *p* < 0.05; ** *p* < 0.01.

To study the changed pathways induced by HFD, an exhaustive KEGG pathway analysis was conducted. Significance was ascertained, using a hypergeometric test with a threshold *p* value ≤ 0.05 to filter out pathways with *p* values higher than 0.05. In detail, the major identified metabolic pathways were platelet activation, arachidonic acid metabolism, ovarian steroidogenesis, biosynthesis of unsaturated fatty acids, ferroptosis, and vitamin B6 metabolism (Tables S3 and S4). The top 20 signaling pathways are shown in the positive (Figure 4A) and negative (Figure 4B) KEGG enrichment plots obtained with MetaboAnalyst. Among these pathways, the vitamin B6 metabolic signaling pathway, arachidonic acid metabolic pathway, biosynthesis of unsaturated fatty acids, platelet activation, serotonergic synapse, ovarian steroidogenesis, and ferroptosis were highly enriched in the HFD rabbit group. Given that PAT is an organ for lipid storage and metabolism, we picked some pathways associated with the lipid cycle for a more detailed analysis of the metabolites (Figure 4C). These metabolites are mainly phospholipids, fatty acids, steroid hormones, and L-methionine, implying that these molecules may be the key molecules in the development of obesity.

## 4. Discussion

Feeding a HFD is the most common method for animal models of obesity, thus HFD continues to be an indispensable method for discovering mechanisms of metabolic syndromes [34]. Similarly, untargeted metabolomics, an effective method to measure metabolites, plays an important role in understanding the physiological functions of metabolites and the potential causes of metabolic disorders [20].

Feeding rabbits with a HFD will result in damage to the normal function of PAT, destruction of the balance between lipid formation and degradation, and overaccumulation of lipids in PAT. The LC-MS/MS metabolite analyses showed that the PAT lipid cycle in rabbits fed a HFD was disturbed, resulting in significant changes in the levels of phospholipids, fatty acids, steroid hormones, and L-methionine (Figure 4C). Concordant with previous studies, heat maps also showed significant differences in metabolites between HFD-fed rats, compared with control (normal diet), and this long-term HFD diet led to the development of obesity-related insulin resistance syndrome in rats [35]. Similarly, feeding mice with a HFD caused a metabolic imbalance that resulted in metabolic disorders, such as insulin resistance and nonalcoholic steatohepatitis [36]. Hematoxylin-eosin staining of PAT showed differences between HFD and SND groups. To further confirm that there is such a difference between them, we used ImageJ to quantify the cells of PAT. The results certainly showed a significant increase in the number of HFD cells and a significant decrease in the area, indicating an increase in cell density in the HFD group, which is consistent with a previous study on human visceral fat [37]. It may cause the absorption of oxygen in lipids to be inhibited, resulting in an imbalance of lipid metabolites and lipid accumulation.

The symptoms of obesity closely resemble the spectrum of metabolic changes in PAT, including phospholipids, fatty acids, steroid hormones, and amino acids, among which phospholipids and lysophosphatides are the most abundant metabolites. Phospholipids are the main components of plasma membranes, including phosphatidylethanolamines (PEs) and phosphatidylcholines (PCs), which are precursors of lysophosphatidylethanolamine (LPEs) and lysophosphatidylcholines (LysoPCs/LPCs), respectively [38]. In our study, PCs and PEs were the most frequently found phospholipids (most of them downregulated in HFD rabbits), whereas levels of all LPCs were reduced. Concerning the 14 PCs and 8 PEs in PAT, some PCs, such as PC (18:4e/20:5) and PC (17:2/22:6), increased in HFD rabbits, which was similar to a previous study in humans, where some PCs were shown to be significantly higher in the obese group than that in the control group [39]. The level of PE (18:2/18:2) was lower in mice fed a HFD than for mice fed a normal diet [40], consistent with our results. Further, the level of LPCs (LPC 15:0, LPC 19:0) decreased in rabbits fed a HFD for 4 weeks, which was partially in agreement with a decrease in plasma in human obesity and Type 2 diabetes [41], and a low-abundance of LPCs in the serum of hyperlipidemic mice fed a HFD [42]. PE is methylated to PC [43]. Changes in phospholipid levels can inhibit calcium ion transport and affect the transfer of phospholipids between the endoplasmic reticulum (ER) and mitochondria, inducing ER stress and mitochondrial dysfunction, which will decrease fatty acid oxidation and acetyl CoA levels [15]. Of course, this is only the speculation made by previous studies that phospholipids may act as a bad metabolite for the characterization of mitochondrial dysfunction. In our next experiments, we will focus on the setting of mitochondria to further improve our research. In addition, an imbalance in the PC/PE ratio will affect the mitochondria-associated ER membranes, leading to an excessive accumulation of sphingomyelin (SM) in the ER, inducing the activation of PKC, inhibiting the activity of AKT and disrupting the energy supply and metabolic homeostasis [16]. SM is produced by group transfer in phosphatidylcholine combined with the associated skeleton, which is closely related to sphingomyelin synthase (SMS). A significant increase in SM may reduce reverse cholesterol transport, increasing the risk of atherosclerosis lesions and other metabolic diseases [17]. Further, as an important signal molecule, reduced levels of LPC combined with some cell-specific G-coupled protein receptors can cause an increase in insulin secretion through glucose stimulation, which will damage β cell function, leading to insulin resistance, stimulate the production of adipocytes, and aggravate the risk of obesity and other diseases [15,44]. These are the factors that may cause insulin resistance. The decrease in LPCs in rabbits from this study may be related to an increase in insulin resistance; thus, LPCs could be considered as potential biomarkers for metabolic diseases caused by obesity due to HFD. Our results here indicate that SM levels were significantly up-regulated in the HFD, compared to the SND rabbit groups, in agreement with reports of obesity and insulin sensitivity in obese adult humans [45] and a study on the plasma metabolic fingerprints of atherosclerosis rabbits [46]. These results suggest that changes in phospholipids levels may reduce insulin sensitivity, lead to insulin resistance, and increase the risk of atherosclerosis.

Arachidonic acid (ARA) and adrenic acid are omega-6 polyunsaturated fatty acids. In the current study, levels of ARA were higher in the HFD than in the SND rabbit group, in agreement with the significant increase in the serum ARA levels in rats fed a HFD [47]. According to our identification results, ARA was the main metabolite of the arachidonic acid metabolic, ovarian steroidogenesis, biosynthesis of unsaturated fatty acids, and ferroptosis metabolism pathways. However, a ‘one-to-many’ type of relationship was pointed out between metabolic pathways that were annotated and identified compounds. ARA matched 18 associated metabolic pathways, which showed that at least ARA was comparatively important for PAT. The most important metabolic pathway for ARA in this study was platelet activation (*p* < 0.01). Under normal circumstances, the release of fatty acids in PAT is strictly controlled to meet energy requirements. Conversely, metabolic disorders cause an excessive release of fatty acids relative to the tissue requirements. It is widely accepted that disturbances in the fatty acid metabolism will lead to increased inflammatory signaling, which is a central factor in insulin resistance [18]. ARA promotes the production of several prostaglandins, which are associated with lipopolysaccharide (LPS)-induced inflammation [48]. It is hypothesized that the increase in ARA is associated with PAT metabolic disorders and may induce inflammation to further produce insulin resistance. Previous studies have shown that the concentration of body fat and adipocytokines in the (HFD + ARA) group was significantly increased after six weeks of induction [49]. Further, excessive levels of ARA can cause oxidative stress and activate pro-inflammatory signals that induce endoplasmic reticulum (ER) stress, leading to insulin resistance [50,51]. Hence, ARA can be used as an indicator of PAT metabolic disorders in obese patients consuming HFD. Endogenous adrenal acid is produced by ARA, and it is mainly oxidized in the peroxisome. Consistent with the results here, the plasma levels of adrenal acid were found to be up-regulated in an adipose hepatitis model, and primarily caused by instability of peroxidase β-oxidation [52]. Docosahexaenoic acid (DHA) and docosapentaenoic acid (DPA) are long-chain omega-3 polyunsaturated fatty acids (PUFAs). The levels of DPA and DHA were significantly elevated in PAT from HFD-fed rabbits, compared to SND-fed rabbits. The higher levels of DPA and DHA in the HFD rabbit group disagreed with the significantly lower levels of DPA and DHA in 12-week-old rats fed a HFD, relative to rats in the control group. Further, a strong positive association existed between the reduced levels of these two metabolites and the insulin sensitivity index [35]. A possible explanation is that a higher concentration of n-3 PUFAs inhibits the release of free fatty acids from PAT [53], which in turn inhibits the inflammatory signaling pathway and decreases the risk of insulin resistance, thus playing a protective role. In addition, DHA and DPA have strong anti-inflammatory effects and can activate peroxisome, thus increasing insulin sensitivity [53,54]. The levels of DPA and DHA in the HFD rabbit group were significantly increased, which may indicate a protective effect. Therefore, rabbits in the HFD group may have insulin resistance and other metabolic syndromes, but they may also produce metabolites, such as DHA and DPA, to protect them against adverse factors. However, no free fatty acids were detected in the plasma/serum of the HFD rabbits; thus, additional research is needed.

Steroid hormones, such as testosterone, 2-hydroxyestradiol, and epitestosterone, were also found in this study. These hormones play vital roles in the production and metabolic function of adipose tissue via hormone receptors. Levels of testosterone, 2-hydroxyestradiol, and epitestosterone were significantly lower in PAT from the HFD than the SND rabbit group. The hormone 2-hydroxyestradiol has strong inhibitory effects on NADPH during lipid peroxidation in rat microsomes. In addition, lipid peroxides depend on specific ions at the initial stage and are strongly inhibited by oxygen absorption [55], which may inhibit the PAT lipid metabolic pathway and cause lipid accumulation. Changes in testosterone were the result of rats being fed HFD to induce insulin resistance [56], strongly suggesting that changes in the rabbit testosterone levels are due to rabbits being fed HFD, inducing insulin resistance. Furthermore, a study showed that testosterone gradually increased in visceral fat rather than in subcutaneous adipose tissue in human females [37]. This result agreed with the significantly increased adipocytes cells and density of PAT in HFD rabbits, relative to SND rabbits. Thus, changes in the 2-hydroxyestradiol and testosterone levels in this study may have led to lipid accumulation by increasing adipocytes cells and the density of PAT and inhibiting the absorption of oxygen by lipid metabolism. However, the specific mechanism of steroid hormones in PAT needs further study.

L-methionine produces methionine, and it functions not only as an essential amino acid, but also as a physiological effector [57]. We found higher levels of L-methionine in HFD than in SND rabbits. The methionine cycle provides methyl units for various reactions, including methylation in lipids. The S-adenosine methionine (SAM) is used as a major methyl donor molecule, and it is synthesized from the essential amino acid methionine [58]. Choline, produced by phosphatidylcholine, and the subsequent substances produced by choline oxidation, such as betaine, can not only help to adjust cell volume, but also act as methyl donors in the homocysteine–methionine (HM) cycle, transporting excess fatty acids to corresponding organelles for metabolism [59]. Methionine supplementation increases the homocysteine (Hcy) concentration and is associated with vitamin B6. Therefore, in our study, one possible explanation is the excessive accumulation of fatty acids in PAT of rabbits fed with a HFD, and the increase in the L-methionine level, thus further increasing the level of Hcy, and finally disturbing the HM cycle. However, previous studies have reported that high circulating Hcy concentrations are related to an elevated risk of atherosclerosis, steatohepatitis and lipid metabolic disturbances [60,61]. Changes in the HM cycle after feeding lean Iberian sows with a HFD were associated with obesity-related diseases and Type 2 diabetes [62], indicating that the higher levels of L-methionine may be related to atherosclerotic diseases and Type 2 diabetes by affecting the HM cycle.

## 5. Conclusions

Histological examination and untargeted metabonomics analysis revealed that rabbits fed a HFD exhibited PAT metabolic disorders, affecting unsaturated fatty acid synthesis, and the arachidonic acid metabolic, ovarian steroidogenesis, and platelet activation pathways. Phospholipids and excessive levels of ARA may cause mitochondrial dysfunction and inflammation, and induce ER stress, leading to insulin resistance. Steroid hormones may inhibit oxygen absorption by increasing the adipocytes cells and density of PAT. L-methionine may increase the risk of Type 2 diabetes and atherosclerosis by affecting the HM cycle. Contrary to previous studies, we found significantly elevated levels of DHA and DPA, which are inversely associated with obesity in both humans and animals. This aspect merits further research. The metabolic changes and biomarkers identified in this study may serve as a foundation for future therapeutic interventions against lipometabolic disorders.

## Figures and Tables

**Table 1:** Metabolites with significant difference were analyzed by LC-MS/MS to identify potential biomarkers of interest.

Metabolites’ Name	Formula	M ^1^	RT ^2^	VIP ^3^	Trend ^4^ and *p* Value (HFD vs. SND)
PC (2:0/16:1)	C26H50NO8P	595.3491	13.322	1.563966765	↓ **
PC (2:0/16:0)	C26H52NO8P	597.36482	13.637	1.678708	↓ *
PC (2:0/16:2)	C26H48NO8P	533.312	13.083	1.257585	↓ **
PC (7:0/8:0)	C23H46NO8P	495.296	13.64	1.709003	↓ **
PC (8:0/8:0)	C24H48NO8P	509.3122	12.994	1.482654	↓ *
PC (14:0e/3:0)	C25H52NO7P	509.3481	15.092	1.797233	↓ **
PC (14:0e/5:0)	C27H56NO7P	537.3799	15.604	1.563913	↓ **
PC (14:0e/15:0)	C37H76NO7P	677.5239	15.723	1.590351	↓ **
PC (15:1/18:2)	C41H76NO8P	741.5327	16.04	1.638059	↓ *
PC (16:0e/13:0)	C37H76NO7P	660.4966	15.72	1.602117	↓ **
PC (16:0e/20:4)	C44H82NO7P	767.5806	16.279	1.665696	↓ *
PC (16:2/18:5)	C42H70NO8P	709.5431	15.675	1.384283	↓ **
PC (17:2/22:6)	C47H78NO8P	815.5423	15.998	1.250684	↑ *
PC (18:1/18:2)	C44H82NO8P	783.5737	16.245	1.218645	↓ *
PC (18:1/19:2)	C45H84NO8P	797.5907	16.194	1.176725	↓ *
PC (18:2/19:2)	C45H82NO8P	795.5781	15.76	2.130609	↓ **
PC (18:4e/20:5)	C46H76NO7P	785.5238	15.649	1.666749	↑ **
PC (18:4e/22:6)	C48H78NO7P	811.5374	15.781	1.790827	↓ **
PC (19:2/18:4)	C45H78NO8P	791.5449	16.305	1.457723	↓ **
PC (20:2/20:3)	C48H86NO8P	835.5957	15.534	1.313735	↓ *
PC (22:3e/18:4)	C48H84NO7P	817.594	15.407	1.361675	↓ **
PC (20:3/20:3)	C48H84NO8P	833.5871	15.567	1.34977	↓ *
LPC 15:0	C23H48NO7P	481.3167	14.571	1.951213	↓ *
LPC 19:0	C27H56NO7P	597.40091	15.577	2.003144	↓ *
PE (2:0/16:2)	C23H42NO8P	491.2647	13.081	1.33104	↓ **
PE (5:0/13:1)	C23H44NO8P	493.2803	13.564	1.864231	↓ **
PE (2:0/18:1)	C25H48NO8P	521.31072	14.223	1.958401	↓ **
PE (17:1/18:1)	C40H76NO8P	729.53193	16.438	2.004323	↓ **
PE (18:2/18:2)	C41H74NO8P	739.5142	16.165	1.086091	↓ *
PE (18:2/20:4)	C43H74NO8P	763.50059	15.487	1.856928	↓ **
PE (20:3/20:4)	C45H76NO8P	789.5234	15.926	1.857655	↑ *
LPE 16:1	C21H42NO7P	451.2696	14.594	1.583397	↓ **
LPE 17:0	C22H46NO7P	467.3011	14.966	1.396921	↓ **
LPE 17:1	C22H44NO7P	465.2855	14.621	2.160675	↓ **
LPE 20:4	C25H44NO7P	501.2857	14.407	1.723687	↑ *
LPS 18:2	C24H44 N O9 P	521.27548	13.894	1.839273	↑ **
LPS 18:0	C24 H48NO9 P	525.30639	14.412	1.545101	↓ *
PG (18:2/20:4)	C44H75O10P	794.51067	15.605	1.039475	↑ *
SM(d19:1/19:0)	C43H87N2O6P	758.6277	15.612	1.201017	↑ *
Arachidonic acid	C20H32O2	304.23985	14.263	1.235326	↑ *
Adrenic acid	C22H36O2	332.27124	14.649	1.732588	↑ *
Docosapentaenoic acid	C22H34O2	330.2556	15.208	2.095414	↑ **
Docosahexaenoic acid	C22H32O2	328.23993	14.209	1.90978	↑ **
Methyltestosterone	C20H30O2	302.22456	14.414	2.348698	↑ **
2-Hydroxyestradiol	C18H24O3	288.17234	10.513	1.100492	↓ **
Epitestosterone	C19H28O2	288.2084	13.648	1.387	↓ *
Cholecalciferol	C27H44O	384.3388	15.714	1.61838	↓ **
4-Pyridoxic acid	C8H9NO4	183.0533	7.482	1.766454	↓ **
L-Methionine	C5H11NO2S	149.0511	1.964	1.568533	↑ *

NOTE: ^1^ m: molecular weight. ^2^ RT: retention time. ^3^ VIP: the importance projection of variables is used to reflect the contribution of quantitative value of each sample to the difference, generally, VIP > 1. ^4^ Trend and *p* value (HFD vs. SND): * *p* < 0.05; ** *p* < 0.01.

## Data Availability

All data generated or analyzed during this study are included.

## Supplementary Materials

The following are available online at https://www.mdpi.com/article/10.3390/ani11082289/s1. Figure S1: Pearson correlation coefficient between positive and negative QC samples. The left is positive ion mode and the right is negative ion mode. Table S1: Total positive metabolites with difference in PAT of rabbits fed either a SND (*n* = 6) or HFD (*n* = 6) was analyzed by LC-MS/MS. Table S2: Total negative metabolites with significant difference in PAT of rabbits fed either SND (*n* = 6) or HFD (*n* = 6) was analyzed by LC-MS/MS. Table S3: KEGG enrichment analysis of differential positive metabolites in PAT of rabbits fed either a SND (*n* = 6) or HFD (*n* = 6). Table S4: KEGG enrichment analysis of differential negative metabolites in PAT of rabbits fed either a SND (*n* = 6) or HFD (*n* = 6).

## Abbreviations

PAT	perirenal adipose tissue
HFD	high-fat diet
SND	standard normal diet
HPLC	high performance liquid chromatography
MS	mass spectrometry
PCA	principal component analysis
PLS-DA	partial least squares discriminant analysis
FC	fold change
QC	quality control
ARA	arachidonic acid
PEs	phosphatidylethanolamines
PCs	phosphatidylcholines
SM	sphingomyelin
SMS	sphingomyelin synthase
ER	endoplasmic reticulum
SAM	s-adenosine methionine
SAH	methyltransferase inhibitor S-adenosylhomocysteine
LPS	lipopolysaccharide
DPA	docosapentaenoic acid
DHA	docosahexaenoic acid
PUFAs	polyunsaturated fatty acids
LPEs	lysophosphatidylethanolamine
LysoPCs/LPCs	lysophosphatidylcholines
Hcy	homocysteine
HM	homocysteine–methionine
LC	liquid chromatography
LC-MS/MS	liquid chromatography-tandem mass spectrometry
TG	triglyceride
KEGG	kyotoencyclopedia of genes and genomes
