# Supplementary figures and images for: Untargeted Metabolomics Analysis Revealed Lipometabolic Disorders in Perirenal Adipose Tissue of Rabbits Subject to a High-Fat Diet
Source: Animals (Basel). 2021 Aug 3;11(8):2289. doi: 10.3390/ani11082289 (PMC8388361; doi:10.3390/ani11082289)

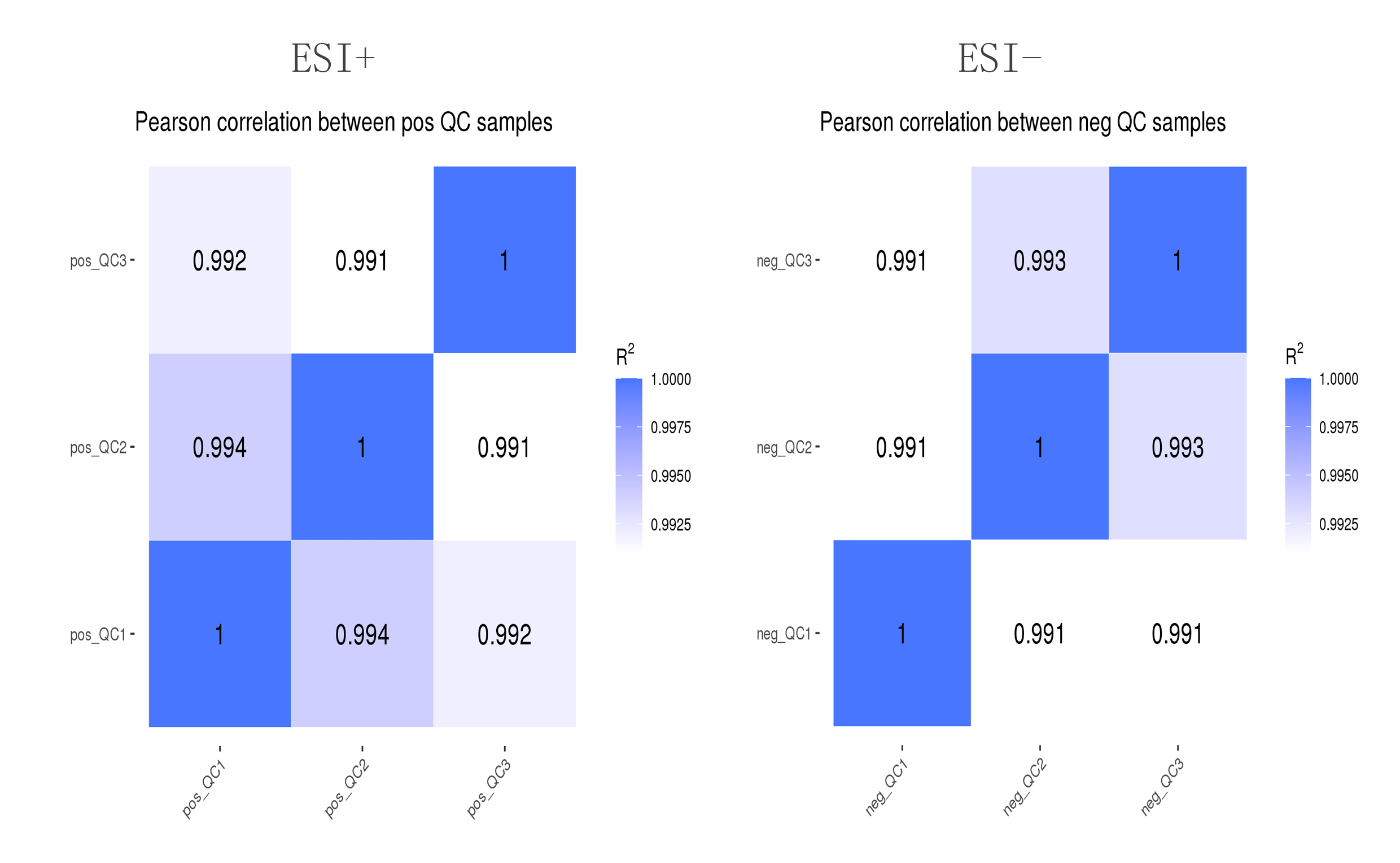

Supplement: Supplementary file 1 [file animals-11-02289-s001.zip › animals-1258100-supplementary/Fig. S1.tif]
